# Comparing Movement Patterns and Physical Function Between Chronic Low Back Pain Patients With Nociplastic and Nociceptive Pain Categories

**DOI:** 10.1002/jsp2.70166

**Published:** 2026-03-01

**Authors:** Erin Archibeck, Nicholas Harris, Patricia Zheng, Aaron Scheffler, Wolf Mehling, Conor O'Neill, Jeffrey Lotz, Grace O'Connell, Jeannie F. Bailey, REACH Investigators, Zehra Akkaya, Prakruthi Amarkumar, Jeannie Bailey, Julia Barylak, Sigurd Berven, Andrew Bishara, Dennis M. Black, Noah Bonnheim, Atul Butte, Joel Castellanos, Jennifer Cummings, Karina Del Rosario, Emilia Demarchis, Sibel Demir‐Deviren, Susan K. Ewing, Adam R. Ferguson, Aaron Fields, Scott M. Fishman, Sergio Garcia Guerra, Fatemeh Gholi Zadeh Kharrat, Xiaojie (Summer) Guo, Misung Han, Trisha Hue, J. Russell Huie, C. Anthony Hunt, Anastasia Keller, Karim Khattab, Roland Krug, Gregorji Kurillo, Feng Lin, Thomas Link, Jeffrey Lotz, John Lynch, Tong Lyu, Rob Matthew, Wolf Mehling, Esmeralda Mendoza, Praveen Mummaneni, Caroline Navy, Conor O’Neill, Jessica Ornowski, Thomas Peterson, Ananya Rupanagunta, Aaron Scheffler, Shalini Shah, Irina Strigo, Naoki Takegami, Abel Torres‐Espin, Salvatore Torrisi, Sachin Umrao, Rohit Vashisht, Joanna Veres, An (Joseph) Vu, Mark Steven Wallace, Lucy Ann Wu, Po‐Hung Wu, Fadel Zeidan, Patricia Zheng, Jiamin Zhou

**Affiliations:** ^1^ Department of Mechanical Engineering UC Berkeley Berkeley California USA; ^2^ Department of Orthopaedic Surgery UC San Francisco San Francisco California USA; ^3^ Department of Epidemiology and Biostatistics UC San Francisco San Francisco California USA; ^4^ Osher Center for Integrative Health, UC San Francisco San Francisco California USA

**Keywords:** biomechanics, chronic low back pain, pain categories, physical function

## Abstract

**Background:**

Individuals with chronic low back pain (LBP) often present with significant physical dysfunction. The underlying cause is difficult to diagnose due to the heterogeneous nature of LBP categories.

**Methods:**

Two hundred and fifty‐six patients were assessed as having either nociceptive (NC) or nociplastic (NP) chronic low back pain using validated surveys, including the PainDETECT Questionnaire and a chronic overlapping pain condition screener. Additional covariates of anxiety, depression, and fear avoidance were evaluated using standard surveys. Physical function was judged objectively using a sit‐to‐stand test (STS; quantified using marker‐less motion capture calculated kinematic scores and movement metrics) and subjectively (PROMIS‐physical function survey). Demographics (age, sex, BMI), psychological factors, and biomechanical outcomes were compared across pain categories using nonparametric statistics and regression modeling.

**Results:**

Compared to the NC group, the NP group was significantly older (NP: 61.0 ± 21.0, NC: 53.5 ± 29.3, *p* = 0.03) and reported higher levels of anxiety (NP: 51.2 ± 17.4, NC: 48.0 ± 13.4, *p* = 0.002) and depression (NP: 49.0 ± 14.7, NC: 41.0 ± 10.8, *p* = 0.009). NP also had worse perceived physical function (PROMIS‐PF) (NP: 39.3 ± 6.9, NC: 42.1 ± 7.3, *p* < 0.001) and slower STS times (NP: 12.5 ± 6.1 s, NC: 12.0 ± 5.8, *p* = 0.03). Despite these differences, the NP group exhibited biomechanical function closer to the healthy control average motion trajectory (K‐score; NP: 77.6 ± 8.1, NC: 75.6 ± 8.1, *p* = 0.03) during the STS task. Regression models evaluating the association between biomechanical variables and pain categories, while adjusting for age, sex, and BMI, identified significant differences between pain categories only for PROMIS‐physical function.

**Conclusion:**

While individuals with nociplastic pain reported lower perceived physical function and exhibited differences in demographic and psychological factors, pain categories were not significant predictors of objective biomechanical measures after adjusting for age, sex, and BMI. However, pain category was a significant predictor of PROMIS‐PF, suggesting that it is more closely associated with perceived functional limitations than with quantitative biomechanical performance.

## Graphical abstract

Are differences in perceived disability between nociceptive and nociplastic cLBP patients reflected in actual movement patterns or in patients' perceived functional ability? Despite worse patient‐reported disability, primarily nociplastic patients did not exhibit worse biomechanical function when compared to primarily nociceptive patients.

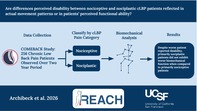

## 1 Introduction

Chronic low back pain (LBP) is multifaceted, with the majority of patients (~90%) diagnosed as having nonspecific LBP due to the absence of clear structural abnormalities [1]. Despite being the highest healthcare expenditure in the United States [2], effective management and treatment for LBP have seen minimal advancement in the past three decades [3], largely due to the complexity of underlying pain mechanisms and the presence of other comorbidities [4]. Patient movement patterns may reflect underlying pain mechanisms, and therefore may aid in diagnosis, treatment selection, and outcomes assessment.

LBP has been proposed to manifest in three pain categories: nociceptive (typically associated with tissue damage), nociplastic (thought to be a result of altered sensory processing), neuropathic (thought to be a result of nerve damage or dysfunction), or a combination of the three [5, 6]. Treatment decisions and outcome expectations are influenced by these pain categories—for example, the presence of nociplastic pain increases the likelihood of poor treatment outcomes and may be better managed with treatments such as cognitive behavioral therapy [7, 8]. Given interactions between pain categories and neuromotor control processes, it is also likely that physical dysfunction and physical response to treatments may differ by pain category. For example, previous research showed that individuals with nociplastic pain reported worse clinical outcomes and less improvement in physical function after physical therapy compared to patients with nociceptive pain [9]. Treatment emphasis also varies depending on the pain category. For nociceptive pain, treatment may emphasize movement quality and adjusting exercises based on pain levels, as well as considering more invasive treatments that target affected tissues such as surgery or injections. In contrast, for nociplastic pain, therapy may focus on encouraging consistent movement through behavioral strategies, such as graded physical activity or mind–body approaches, with less attention to movement mechanics [10, 11, 12]. It is important to note that the nociceptive, neuropathic, and nociplastic categories represent one current way of classifying back pain, and these pain categories may be used to identify the treatments that are most likely to be helpful. Current guidelines often recommend a personalized diagnostic approach because pain etiology can be highly individualized, and thus elusive on a patient‐to‐patient basis [13].

Pain categories for chronic pain are widely recognized as being influenced by biopsychosocial factors, such as age, sex, and body mass index (BMI) [9, 14]. Age is linked to the prevalence and severity of LBP [15, 16, 17], and there is a well‐known sexual dimorphism in chronic pain experience [18, 19, 20]. Additionally, there is a concurrence of higher BMI and chronic pain [21, 22], and mental health factors, such as increased anxiety and depression that coincide with chronic pain, particularly nociplastic pain [23, 24]. Concerning motion, while sex‐based and age‐based differences in movement quality have been observed [25], the impact of how they affect the relationship between pain categories and resulting motion patterns is not understood. Overall, there remains a gap in understanding a potential association between biomechanical function and pain categories, including how patient factors may influence such a relationship.

Therefore, the objective of this study was to determine whether biomechanical function differs across pain categories in patients with LBP, accounting for demographic and psychological factors. Specifically, we focused on the two predominant pain categories observed in the LBP cohort, nociceptive and nociplastic pain, and recorded both self‐reported physical function and quantitative biomechanical measures. Patients classified with neuropathic pain were excluded due to their low representation (*n* = 8), as well as to avoid potential confounding that could occur by grouping them with another category for comparison. We hypothesized that differences in pain categories would be reflected either in actual movement patterns or in patients' perceived functional ability. Understanding how underlying pain categories relate to patient‐specific biomechanical impairment for those with chronic LBP will help inform effective treatment selection.

## 2 Methods

### 2.1 Patient Selection

A subset of data was obtained from the Institutional Review Board‐approved (IRB #20204648) Longitudinal Clinical Cohort for Comprehensive Deep Phenotyping of Chronic Low‐Back Pain Adults Study (ComeBACK) study [26]. ComeBACK is a longitudinal cohort study focused on adults with chronic LBP. Eligible participants had experienced LBP for more than 3 months, affecting over 50% of days. Recruitment for the ComeBACK study took place among four participating clinical sites across the University of California system and involved recruitment through their respective patient populations—especially those who were presently or previously involved in treatment for their LBP at any of the clinical sites. Extensive data was obtained from the ComeBACK study on demographics, pain, and biomechanical function for 256 participants with LBP categorized in the nociceptive and nociplastic groups. These 256 participants represent a sub‐group of the broader study (*n* = 450). Patients were not included if they had incomplete or low quality motion analysis data relevant to this study (*n* = 127) or fell into either the neuropathic (*n* = 8) or mixed (*n* = 53) groups for purposes of maintaining a streamlined analysis and minimizing potential confounding. To help avoid selection bias, no exclusions were made on the basis of outcome measures or other post hoc considerations. Additionally, 60 control participants from the ComeBACK study served as normative references for the Kinematic Composite Score (K‐score) Algorithm. The control group was age‐matched with the LBP group and had no reported LBP in the preceding 24 months. Exclusion criteria for both the control and LBP groups encompassed significant comorbidities, contraindications for MRI, a history of spine or lower extremity surgery, referred pain, BMI exceeding 35, or an inability to walk unaided.

### 2.2 Pain Category

#### 2.2.1 Neuropathic and Mixed Screening

For this analysis, the pain detect questionnaire (PD‐Q) was used to categorize and ultimately exclude patients who scored as having either “possible” or “likely” neuropathic pain [27]. The PD‐Q survey consists of nine questions, with a total score that ranges from −1 to 38. Examples of questions administered included: “Do you suffer from a burning sensation (e.g., stinging nettles),” or “Do you suffer from a sensation of numbness?” Answers are rated by the patient as either “Never,” “Hardly Noticed,” “Slightly,” “Moderately,” “Strongly,” or “Very Strongly,” and responses are scored from 0 (*Never*) to 5 (*Very Strongly*). A higher total score indicates greater neuropathic pain intensity. PD‐Q scores that were less than or equal to 12 were labeled as “Non‐Neuropathic.” PD‐Q scores greater than 12 and less than 19 were labeled as “Possibly Neuropathic,” and PD‐Q scores greater than or equal to 19 were designated as “Neuropathic.” The “Neuropathic” pain group was excluded due to its small sample size (*n* = 8), which limited statistical power and reduced the reliability of comparisons across groups. A mixed pain group (*n* = 53), constituting patients that fell into both the neuropathic and nociplastic categories, was also excluded due to concerns that it would have introduced bias and unstable estimates.

#### 2.2.2 Nociplastic

To further categorize the large group of patients who were identified as non‐neuropathic from the PD‐Q survey (*n* = 256), nociplastic patients were identified using the results from two questionnaires aimed at evaluating the prevalence of non‐lumbar chronic pain and pain syndromes. The first questionnaire was the widespread pain inventory (WPI) from the BACPAC minimum dataset [28], and the other questionnaire was a standardized screening form based on the self‐report version of the Charlson comorbidity index [29]. The WPI asked patients if they had chronic pain in any of the following areas: Head or Face, Left or Right Hand/Arm/Shoulder, Left or Right Buttock/Leg/Foot, Chest/Abdomen/Pelvis, Neck or Upper Back. Excluding pain that is associated with cLBP (Left or Right Buttock/Leg/Foot), the total number of non‐lumbar regional pain sites that a participant identifies (0–5) is used to help inform the nociplastic designation. Similarly, the self‐report version of the Charlson comorbidity index included 21 questions about comorbidities, with a subset of eight chronic pain conditions that are used to help evaluate if a participant may have nociplastic pain. The relevant pain conditions were as follows: Endometriosis, Fibromyalgia, Interstitial Cystitis/Irritable Bladder, Irritable Bowel Syndrome, Migraine or Chronic Headache, Chronic Prostatitis, Temporomandibular Joint Syndrome, or Vulvodynia. If the participant indicated they had ever received a diagnosis (yes/no) for each of the above conditions, this would be added to a tally for chronic overlapping pain conditions (COPC) with a minimum of zero conditions and a maximum of eight. The tally was tracked for the participants over the course of the study and follow‐up period, with the final designation and count of conditions being made at the conclusion of study activities. Among the patients who had not already been classified as having neuropathic pain, patients with two or more COPC's, or two or more non‐lumbar regional pain sites, were assigned to the nociplastic pain category, and patients with less than two COPC's and non‐lumbar regional pain sites were excluded from the nociplastic category.

#### 2.2.3 Nociceptive

Patients who did not fall into either the nociplastic (> 2 COPCs or non‐lumbar regional pain sites), neuropathic/possibly neuropathic (PD‐Q score > 12), or mixed pain designations were classified as nociceptive.

Finally, due to the complex factors influencing mixed pain, the category was excluded to simplify the analysis and focus on primary categories.

### 2.3 Biomechanical Function

LBP‐related disability is commonly measured using quantitative, yet subjective, self‐reported questionnaires such as the Patient‐Reported Outcomes Measurement Information System (PROMIS) Physical Function (PF) [30]. The PROMIS‐PF Short Form 6b was used, where a higher PROMIS‐PF score indicates greater physical functioning (possible score range: 6–30). Scores were normalized using the PROMIS T‐score system, where 50 is the mean and 10 is a standard deviation. Additionally, quantitative objective measures of physical function were captured using a markerless motion capture system (Azure Kinect, Microsoft). The system recorded joint positions (30 Hz) at key trunk (neck, shoulders, mid‐spine, base of spine) and lower extremity (hips, knees, ankles) landmarks. Participants performed five unassisted sit‐to‐stand (STS) transitions from a standard‐height chair (18 in.). STS was chosen as an easily repeatable functionally relevant task requiring coordinated trunk and lower limb motion [31]. Selection of STS as opposed to other movements was also limited by the biomechanical data that was collected as part of the broader ComeBACK study. Other movements were tested during the PT exam where the STS testing took place; however, they were not recorded for motion analysis.

In this project, a time outcome was included based on the traditional five‐times STS assessment, performed by a physical therapist [32]. It is important to note that the biomechanical assessment of STS motion analysis was based on a separate protocol for STS where patients had their arms at their sides and were advised to come to a quiet pause in between transfers.

From the motion analysis, peak Sagittal Vertical Axis (SVA) was measured as the maximum anterior displacement of the shoulder relative to the hip, normalized by subject height. Poor sagittal alignment has been shown to be linked with back pain and to correlate well with poor outcomes on measures such as ODI, as well as measures of physical performance such as the one leg standing test or 6‐min gait [33]. Maximum torso velocity was computed in the anterior (horizontal) and superior (vertical) directions by differentiating the position of the center of mass of the torso over time and identifying the peak velocity in each direction. Finally, the maximum torso velocity was computed in the anterior (horizontal) direction by differentiating the torso's position (center of mass) over time and identifying the peak velocity in the specified direction. Velocity was normalized by the square root of the product of gravitational acceleration and the individual's leg length.

Additionally, a composite metric, the K‐score, provides a single value representing the overall deviation of a motion trajectory from healthy controls, rather than focusing on an isolated metric [34]. To measure the K‐score of the trunk (torso landmarks) and full‐body (all landmarks), principal component analysis (PCA) was used to quantify movement control [31]. Then, a Generalized Procrustes Analysis was used to standardize data across participants by aligning PCA‐transformed positions to a reference frame. Averaged data from the control group was used as the reference. The K‐score quantified the total deviation across every time point from the control average, adjusted for movement speed. Scores were transformed such that 100 represented the control trajectory.

### 2.4 Demographic Factors

Age, sex, and BMI were recorded for every individual. Further, anxiety, depression, and fear avoidance levels were approximated by survey administration. Anxiety was captured using the Patient‐Reported Outcomes Measurement Information System (PROMIS) Anxiety Short Form, which is scored from 4 to 20 and then normalized using the PROMIS T‐score system. A higher score indicates greater levels of anxiety. Concerning depression, the PROMIS Depression Short Form is scored from 4 to 20 and then normalized using the PROMIS T‐score system. A higher T‐score indicates greater levels of depression. Finally, fear avoidance was measured using the Fear Avoidance Beliefs Questionnaire—Physical Activity (FABQ‐PA). The FABQ‐PA form focuses on the respondent's beliefs about their pain and its relationship to physical activity. The scores range from 0 to 24, with higher scores meaning greater fear avoidance beliefs. For the ComeBACK study, “High” fear avoidance beliefs was a score of > 13 on the physical activity subform.

### 2.5 Outcomes and Statistics

A chi‐squared test was performed to determine if there were significant differences in sex frequencies between the two pain categories. The demographic, psychological, and biomechanical variables were compared across two pain categories (nociceptive and nociplastic) using the Kruskal–Wallis test due to the nonparametric nature of the data. A *p* < 0.05 was defined as statistically significant, and an effect size (*η* ^2^) between 0.01–0.06 and 0.06–0.14 was considered small and moderate, respectively. Subsequently, multiple linear regression models (Equation 1) were constructed to examine how pain categories and demographic factors predict or explain variation in biomechanical function. Given prior work highlighting sex‐based differences in biomechanical function, a secondary regression analysis was constructed (Equation 2) to include an interaction term to assess whether the effect of pain category on biomechanics differs by sex. Psychological factors such as anxiety, depression, and fear avoidance were excluded from this analysis, as previous research has indicated that they represent downstream consequences of the underlying pain category rather than primary determinants of biomechanical differences [35, 36, 37].

(1) Biomechanical variable~Pain category+Sex+Age+BMI

(2) $$\begin{array}{c} \text{Biomechanical Variable}\sim \text{Pain category}+\mathrm{Sex}+\mathrm{Age}\\ +\mathrm{BMI}+\text{Pain Category}:\mathrm{Sex} \end{array}$$

## 3 Results

### 3.1 Demographic and Psychological Factors

**FIGURE 1:**
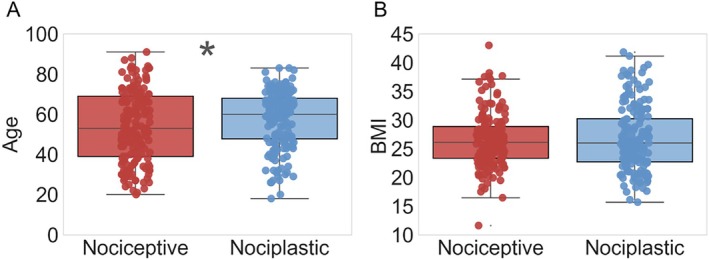
Distribution of age (A) and BMI (B) across pain categories. *p < 0.05.

This study included 256 patients with LBP. Within this sample, 117 were males, 58% (*n* = 68) exhibited nociceptive pain, and 42% (*n* = 49) exhibited nociplastic pain (Table 1). Among the 139 females with LBP, 42% (*n* = 59) exhibited nociceptive pain, while 58% (*n* = 80) exhibited nociplastic pain. A statistically significant difference was observed in the distribution of sex across the pain categories (*χ* ^2^ = 10, *p* = 0.007), with a notably higher proportion of females in the nociplastic group. Further, there was a statistical significance in age between the nociceptive (53.5 ± 29.3 years) and nociplastic groups (61.0 ± 21.0 years, *p* = 0.03; Figure 1A), but no significant difference in BMI (*p* = 0.92, Figure 1B).

**TABLE 1:** Demographics for nociceptive and nociplastic pain categories.

	Sex	Age	BMI
Nociceptive	54% male	53.5 ± 29.2	26.2 ± 5.7
Nociplastic	37% male	61 ± 21.0	25.4 ± 7.6
*p‐*value	0.007	0.03	0.93

**FIGURE 2:**
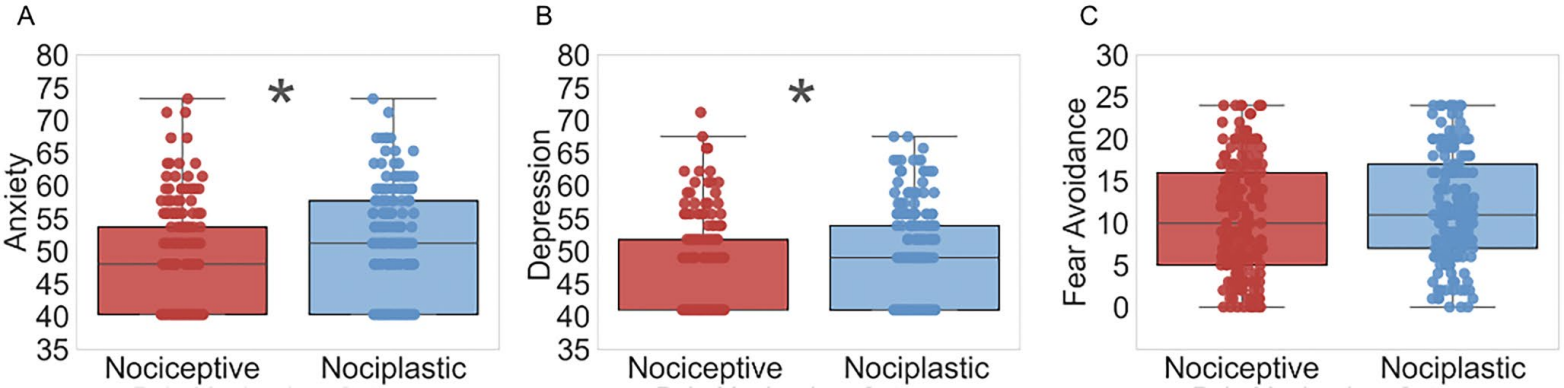
Distribution of reported anxiety (A), depression (B), and fear avoidance (C) levels across pain categories. *p < 0.05.

Concerning psychological factors, the nociplastic group exhibited significantly higher anxiety (51.2 ± 17.4) compared to the nociceptive group (48.0 ± 13.4, *p* = 0.002). Similarly, depression was greater in the nociplastic group (49.0 ± 14.7) when compared to the nociceptive group (41.0 ± 10.8, *p* = 0.009; Figure 2). There was no difference in fear avoidance between the two pain categories (*p* = 0.11).

### 3.2 Biomechanical Variables

**FIGURE 3:**
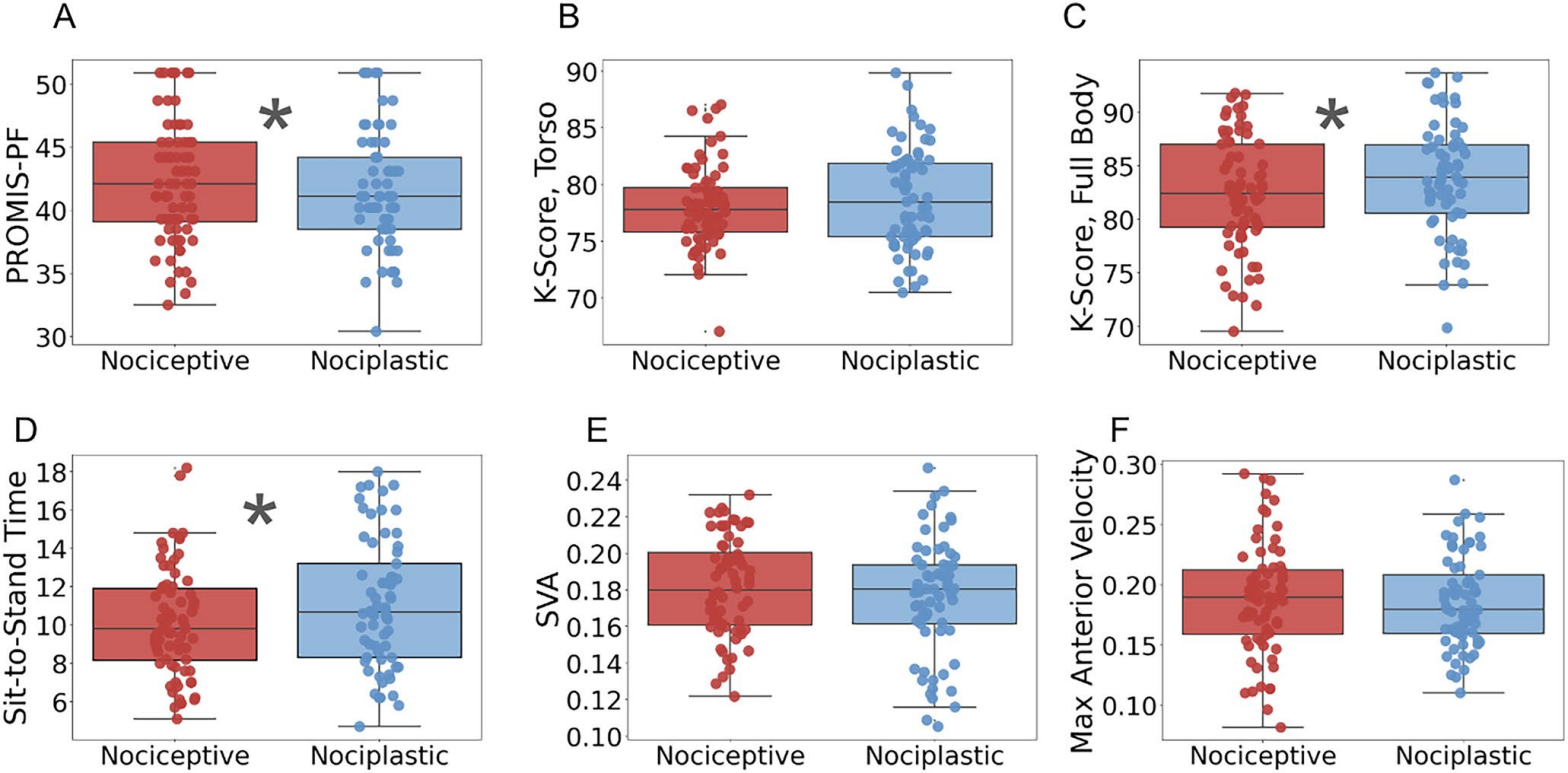
Distributions of PROMIS‐PF scores (A), torso K‐scores (B), full body K‐scores (C), sit‐to‐stand times (D), SVA values (E), and maximum anterior velocity (F) across pain categories. *p < 0.05.

Physical function based on PROMIS‐PF scores was lower for the nociplastic group (40.2 ± 4.6 vs. 42.1 ± 6.4 for nociceptive, *p* = 0.04, *η* ^2^ = 0.02; Figure 3A). The full‐body K‐score for the nociplastic group (83.3 ± 7.0) was statistically greater than the nociceptive group (80.9 ± 7.9, *p* = 0.04, *η* ^2^ = 0.02; Figure 3C); however, the relative difference was less than 5%. The total STS time was 12% greater for the nociplastic group (11.0 ± 3.6 s) than the nociceptive group (9.8 ± 3.7 s, *p* = 0.04, *η* ^2^ = 0.02; Figure 3D). There was no significant difference in torso K‐score between individuals with nociplastic pain (77.7 ± 7.9) and those with nociceptive pain (76.3 ± 4.4, *p* = 0.08; Figure 3B). Further, there were no significant differences in SVA or maximum torso anterior velocity between the two pain groups (*p* > 0.5; Figure 3E,F, respectively).

### 3.3 Relationship Between Variables

Separate multiple variable regression models were conducted for each physical function outcome. In these models, the physical function variable served as the outcome, while pain category and demographics (age, sex, BMI) were included as predictors.

In the initial regression models (Eq. 1), which evaluated the association between pain category (primary variable of interest) and six biomechanical outcomes—adjusting for sex, age, and BMI—no significant predictors were identified for the full‐body K‐score, STS time, or maximum anterior velocity outcomes (Table 2). While the models for torso K‐score and SVA included other significant covariates, the pain category itself was not a significant predictor (*p* = 0.36 and 0.25, respectively). However, pain categories were a significant predictor for PROMIS‐PF (*p* < 0.001), indicating that pain classification may be more closely linked to self‐reported function than to observed biomechanical metrics. Specifically, the Kruskal–Wallis analysis noted that nociplastic patients reported lower PROMIS‐PF scores.

**TABLE 2:** Biomechanical variables (PROMIS‐PF, K‐score torso, K‐score full body, sit‐to‐stand time, SVA, max anterior velocity) across pain categories.

	Nociceptive	Nociplastic	*p*
PROMIS‐PF	42.1 ± 6.4	40.2 ± 4.6	0.04
K‐score torso	76.3 ± 4.4	77.7 ± 7.9	0.08
K‐score full body	80.9 ± 7.9	83.3 ± 7.0	0.04
Sit‐to‐stand time	9.8 ± 3.7	11.0 ± 3.6	0.04
SVA	0.2 ± 0.04	0.2 ± 0.03	0.55
Max anterior velocity	0.2 ± 0.04	0.2 ± 0.06	0.89

To assess whether the effect of pain category differed by sex, interaction terms between pain category and sex were introduced in a second set of models (Eq. 2). The interaction term was not significant in any outcome model, suggesting that the relationship between pain category and biomechanical function does not differ between males and females.

## 4 Discussion

In this study, we aimed to determine if there were differences in biomechanical function based on pain classification, while considering other factors that may interfere with both pain and function. Biomechanical function was assessed using self‐reported and objective measures, including PROMIS‐PF scores, STS time, maximum velocity, peak vertical sagittal alignment, and overall movement quality (K‐score). When initially examining how individual measures varied between nociceptive and nociplastic pain categories, the nociplastic group was statistically older and reported higher levels of anxiety and depression, along with lower perceived physical function (PROMIS‐PF) and slightly slower STS times. However, the quality of the full‐body biomechanical motion of the nociplastic group (K‐score) during the STS task was higher (more similar to controls) than the nociceptive group. Subsequently, multiple linear regression models were developed to assess the association between these variables and pain categories, while adjusting for potential confounders such as age, sex, and BMI (Table 3). Upon conducting these analyses, the pain category emerged as a significant predictor only for PROMIS‐PF, suggesting that pain category significantly influences individuals' self‐reported physical function. However, pain categories did not impact quantitative biomechanical measures.

**TABLE 3:** Multiple linear regression models for the six biomechanical variables (PROMIS‐PF, torso K‐score, full‐body K‐score, sit‐to‐stand time, SVA, and max anterior velocity).

Variable	Coefficient	*p*	95% Confidence interval	Standardized coefficient
PROMIS—PF ~ Pain category + Sex + Age + BMI				
Pain category	−1.2736	0.000	[−1.9004, −0.6468]	−0.0577
Age	−0.0252	0.201	[−0.0640, 0.0135]	−0.0011
Sex	−1.2232	0.058	[−2.4858, 0.0394]	−0.0554
BMI	−0.2396	0.000	[−0.3573, −0.1219]	−0.0109
Torso K‐score ~ Pain category + Sex + Age + BMI				
Pain category	0.2993	0.358	[−0.3416, 0.9402]	0.0136
Age	−0.0068	0.735	[−0.0466, 0.0330]	−0.0003
Sex	1.3856	0.003	[0.1242, 2.6470]	0.0628
BMI	−0.1815	0.032	[−0.3011, −0.0619]	−0.0082
Full‐body K‐score ~ Pain category + Sex + Age + BMI				
Pain category	0.4533	0.323	[−0.4495, 1.3562]	0.0205
Age	0.0109	0.701	[−0.0451, 0.0670]	0.0005
Sex	1.4439	0.111	[−0.3330, 3.2208]	0.0654
BMI	0.0353	0.680	[−0.1332, 0.2037]	0.0016
Sit‐to‐stand time ~ Pain category + Sex + Age + BMI				
Pain category	0.2223	0.853	[−2.1300, 2.5747]	0.0101
Age	0.1140	0.122	[−0.0305, 0.2584]	0.0052
Sex	2.8882	0.232	[−1.8537, 7.6301]	0.1308
BMI	−0.3281	0.148	[−0.7735, 0.1173]	−0.0149
SVA ~ Pain category + Sex + Age + BMI				
Pain category	0.0026	0.248	[−0.0018, 0.0070]	0.0001
Age	0.0001	0.450	[−0.0002, 0.0004]	0.0000
Sex	−0.0133	0.004	[−0.0222, −0.0043]	−0.0006
BMI	0.0001	0.747	[−0.0007, 0.0010]	0.0000
Max anterior velocity ~ Pain category + Sex + Age + BMI				
Pain category	0.0019	0.595	[−0.0050, 0.0088]	0.0001
Age	0.0000	0.951	[−0.0004, 0.0004]	0.0000
Sex	−0.0012	0.863	[−0.0151, 0.0127]	−0.0001
BMI	−0.0001	0.904	[−0.0014, 0.0012]	−0.0000

When analyzing the pairwise results, the differences found in demographic and psychological differences in the nociplastic and nociceptive groups largely align with previous epidemiological studies. Nociplastic pain is characterized by changes in the nociceptive processing, creating hypersensitivity to stimuli [38]. Therefore, the prevalence of depression and anxiety is a hallmark symptom among those with chronic nociplastic pain, such as fibromyalgia [39]. Our results confirm prior findings, with the predominantly nociplastic pain category showing elevated anxiety and depression levels compared to the predominantly nociceptive group (Figure 2). Additionally, females are approximately 1.5–2 times more likely to have nociplastic pain than males [35], which was also reflected by our results (Table 1). Interestingly, findings from our study showed that patients with nociplastic pain were older in our study population. There is minimal research on the relationship between age and pain categories. Previous studies have suggested that nociplastic pain, particularly in fibromyalgia, often develops at a younger age and may have a genetic component [40, 41].

Concerning biomechanical function, the predominantly nociplastic group moved slower through the STS activity and reported worse physical function, yet their full‐body K‐scores appear to indicate better full‐body movement quality during STS (Table 3). These results highlight an interesting disconnect between perceived disability (PROMIS‐PF) and actual biomechanical quality (STS K‐score). One potential explanation is that individuals in the nociplastic group move slower due to an amplified perception of disability, as reflected by the PROMIS‐PF score [42]. However, despite this heightened perception of physical inability, the nociplastic group's pain is unlikely to be attributable to identifiable musculoskeletal damage or inflammation, as the pain was less localized. Therefore, while patients with nociplastic pain may move more cautiously, our results indicate that their actual posture may be less impaired. In contrast, the nociceptive group is assumed to have structural and mechanical causes of pain, which may be contributing to impaired biomechanical responses due to compensatory strategies [43]. It is worth noting, identifying specific structural and mechanical causes is often difficult for chronic low back patients. Other kinematic measures that are often reported for biomechanical function, such as SVA and maximum velocity, did not differ significantly between the two pain groups (*p* > 0.5). Therefore, while we can infer that individuals with nociplastic pain move more cautiously (with slower total time) and nociceptive pain generally demonstrates lower movement quality, the differences are modest and not reflective across all variables. Lastly, the differences we found in patient reported disability (PROMIS‐PF) and biomechanical function may indicate a need to reconsider how effective subjective questionnaires are at evaluating physical function and recovery.

When adjusting these findings by applying multiple regression models to adjust for age, sex, and BMI, pain category was not a predictor for the quantitative biomechanical measures (K‐score, SVA, STS times), yet showed significance for perceived physical function (PROMIS‐PF) (Table 3). While pain categories might impact motor activity and control [11], the results highlight that pain categories may not affect measurable physical performance, but do play a role in how individuals perceive their own physical abilities. Clinically, this can inform treatment strategies by emphasizing the importance of addressing perceived function through interventions that focus on managing psychosocial factors and using cognitive, behavioral or functional therapies to help with exposing the patient to more movements, rather than passive therapies in individuals with nociplastic pain, reaffirming previous research [44]. As a result, motion‐based diagnostics may be of limited value when trying to categorize patients into pain types. It is critical to question the use of how pain classifications are driving clinical and physical therapy decisions [10], potentially incorporating comprehensive biomechanical function metrics and additional demographic information to improve patient‐specific treatment.

This study was not without its limitations. Effect sizes were generally moderate to small, where applicable. Also, while the individual questionnaires used for classifying pain in our study are validated, using them to classify a patient's pain category, as we have done in this paper, has not been validated. There are currently no validated approaches for classifying a patient's pain category that is done exclusively via questionnaire, and as such most current guidelines for pain classification recommend a more systematic approach that integrates detailed history taking with findings from physical exam and/or imaging [10, 45]. For nociplastic pain, while the questionnaires targeting non‐lumbar pain & pain syndromes may help identify the presence of nociplastic pain, current IASP clinical guidelines still recommend a more thorough investigation assessing features such as hypersensitivity in the low back region [44]. The nociceptive designation was performed through a process of elimination and therefore is reliant on the validity of the other categories. Finally, our biomechanical evaluation only looked at one movement (STS) for which there was quality data, whereas a comprehensive analysis would include multiple movements in various planes, with a tailored exam and data collection specifically for biomechanical analysis. The STS movement itself would have benefitted from more in‐depth measuring of pain, such as movement‐evoked pain during the STS activity and specifically what aspects of the movement are most pain provoking. Future research will explore movement‐evoked LBP as it relates to symptoms and potential mechanisms underlying patient‐experienced pain. Finally, we excluded mixed pain from our analysis due to concerns about its complex etiology limiting the precision of our analysis, but future work would benefit from including this group when data quality allows it, as they comprise a nontrivial portion of LBP patients and thus merit further research.

Overall, pain categories are complex, often overlap, and are difficult to define. The primary original goal of this work is to leverage movement patterns and overall physical function to identify underlying pain categories, ultimately leading to more accurate and targeted clinical therapy strategies. To begin exploring the relationship between movement and pain classifications, this research identified pain categories through patient questionnaires and assessed whether the pain classifications were associated with differences in biomechanical function. Although differences were observed, the effect size was small, and overall biomechanical function was not dramatically distinct between the two primary pain categories (nociplastic and nociceptive). Further, pain categories were not significant predictors of objective biomechanical measures after adjusting for age, sex, and BMI. However, the pain category was a significant predictor of PROMIS‐PF, suggesting that it is more closely associated with perceived functional limitations than with quantitative biomechanical performance. This suggests that pain categories are more closely associated with perceived functional limitations than with objectively measured biomechanical performance.

## Author Contributions

E.A., N.H., G.O.C., and J.F.B., take responsibility for the integrity of the work as a whole, from inception to finished article. E.A., N.H., and J.F.B. wrote and modified the manuscript. E.A., N.H., and REACH Investigators collected data. P.Z., W.H., C.O.N., and J.L. advised on the analysis plan and clinical interpretation of results. A.S. performed statistical analysis. All authors read and approved the manuscript.

## Funding

This work was supported by the National Science Foundation, DGE 2146752 and National Institutes of Health, U19AR076737, R01AR081324.

## Conflicts of Interest

J.F.B. has stock options with Bioniks, Limited Liability Company. G.O. has received compensation as a member of the scientific advisory board of AT Dev Inc. and owns stock in the company. The other authors declare no conflicts of interest.

## Data Availability

The data that support the findings of this study are available from the corresponding author upon reasonable request.
